# A Prioritized and Validated Resource of Mitochondrial Proteins in *Plasmodium* Identifies Unique Biology

**DOI:** 10.1128/mSphere.00614-21

**Published:** 2021-09-08

**Authors:** Selma L. van Esveld, Lisette Meerstein‐Kessel, Cas Boshoven, Jochem F. Baaij, Konstantin Barylyuk, Jordy P. M. Coolen, Joeri van Strien, Ronald A. J. Duim, Bas E. Dutilh, Daniel R. Garza, Marijn Letterie, Nicholas I. Proellochs, Michelle N. de Ridder, Prashanna Balaji Venkatasubramanian, Laura E. de Vries, Ross F. Waller, Taco W. A. Kooij, Martijn A. Huynen

**Affiliations:** a Center for Molecular and Biomolecular Informatics, Radboud Institute for Molecular Life Sciences, Radboudumc, Nijmegen, the Netherlands; b Radboud Center for Mitochondrial Medicine, Radboudumc, Nijmegen, the Netherlands; c Radboud Institute for Health Sciences, Radboudumc, Nijmegen, the Netherlands; d Department of Medical Microbiology, Radboudumc Center for Infectious Diseases, Radboud Institute for Molecular Life Sciences, Radboudumc, Nijmegen, the Netherlands; e Department of Biochemistry, University of Cambridge, Cambridge, United Kingdom; f Theoretical Biology and Bioinformatics, Science for Life, Utrecht University, Utrecht, the Netherlands; g Laboratory of Molecular Bacteriology (Rega Institute), Department of Microbiology, Immunology and Transplantation, KU Leuven, Leuven, Belgium; University at Buffalo

**Keywords:** *Plasmodium*, Bayesian data integration, mitochondria

## Abstract

*Plasmodium* species have a single mitochondrion that is essential for their survival and has been successfully targeted by antimalarial drugs. Most mitochondrial proteins are imported into this organelle, and our picture of the *Plasmodium* mitochondrial proteome remains incomplete. Many data sources contain information about mitochondrial localization, including proteome and gene expression profiles, orthology to mitochondrial proteins from other species, coevolutionary relationships, and amino acid sequences, each with different coverage and reliability. To obtain a comprehensive, prioritized list of Plasmodium falciparum mitochondrial proteins, we rigorously analyzed and integrated eight data sets using Bayesian statistics into a predictive score per protein for mitochondrial localization. At a corrected false discovery rate of 25%, we identified 445 proteins with a sensitivity of 87% and a specificity of 97%. They include proteins that have not been identified as mitochondrial in other eukaryotes but have characterized homologs in bacteria that are involved in metabolism or translation. Mitochondrial localization of seven Plasmodium berghei orthologs was confirmed by epitope labeling and colocalization with a mitochondrial marker protein. One of these belongs to a newly identified apicomplexan mitochondrial protein family that in P. falciparum has four members. With the experimentally validated mitochondrial proteins and the complete ranked P. falciparum proteome, which we have named PlasmoMitoCarta, we present a resource to study unique proteins of *Plasmodium* mitochondria.

**IMPORTANCE** The unique biology and medical relevance of the mitochondrion of the malaria parasite Plasmodium falciparum have made it the subject of many studies. However, we actually do not have a comprehensive assessment of which proteins reside in this organelle. Many omics data are available that are predictive of mitochondrial localization, such as proteomics data and expression data. Individual data sets are, however, rarely complete and can provide conflicting evidence. We integrated a wide variety of available omics data in a manner that exploits the relative strengths of the data sets. Our analysis gave a predictive score for the mitochondrial localization to each nuclear encoded P. falciparum protein and identified 445 likely mitochondrial proteins. We experimentally validated the mitochondrial localization of seven of the new mitochondrial proteins, confirming the quality of the complete list. These include proteins that have not been observed mitochondria before, adding unique mitochondrial functions to P. falciparum.

## INTRODUCTION

Although all mitochondria evolved from a single endosymbiotic event (1), they display a large variety among the eukaryotes. For example, while in most species the mitochondrial organelle retains its genome, the number of proteins encoded varies from 100 in Andalucia godoyi (2) to three in myzozoan species like Plasmodium falciparum that encode only cytochrome *b*, cytochrome *c* oxidase subunits 1 and 3, and two fragmented rRNAs (3). Moreover, mitochondrion-like organelles of a variety of anaerobic species have lost the organellar genome altogether (4). Also, mitochondrial proteomes vary broadly in size, ranging from, e.g., ∼1,500 different proteins in mammalian mitochondria (5) to a few members of only a single biochemical pathway found in some mitosomes (6). Within this variety, the P. falciparum mitochondrion, based on the size of its genome, the size of the nuclear genome, and the complexity of its oxidative phosphorylation, is predicted to have a relatively small proteome. Although several mitochondrial pathways have already been identified in P. falciparum (7), and databases like PlasmoDB contain valuable information about mitochondrial localization of individual proteins (8), a complete resource that weighs and combines all the relevant information about possible mitochondrial localization is lacking.

*Plasmodium* mitochondria are particularly interesting because their function is variable between different life cycle stages. In the sexual blood stage, functional oxidative phosphorylation is essential for colonization and development inside the mosquito hosts (9, 10), while in asexual blood stages, the only essential function of the respiratory chain is to recycle ubiquinone for pyrimidine biosynthesis (11). This is further highlighted by the observation that in asexual blood-stage parasites, no cristae are detectable in the mitochondrial membrane, while in gametocyte mitochondria, crista-like structures that typically accumulate respiratory chain complexes are observed (10, 12). A recent complexome profiling study demonstrated that the level of complex V components in asexual blood-stage parasites was only 3% of the level in gametocytes (12). Another intriguing aspect of mitochondria in *Plasmodium* and closely related species is that they have a rather unique FeS cluster assembly pathway (13). From this pathway, the frataxin protein, an FeS assembly protein that occurs even in mitosomes (14), appears to be missing in *Plasmodium* (15).

Knowledge of the mitochondrial proteome could provide viable targets for the development of new drugs against this deadly parasite, as apicomplexan mitochondrial proteomes contain unique proteins that are not present in the animal hosts (16). Recently for example, an unusual prohibitin-like protein, PHBL, was identified in Plasmodium berghei that might provide a transmission-blocking drug target, as it is unique to Myzozoa and *phbl*^−^ parasites fail to transmit (17). Furthermore, respiratory protein complex III has proven to be a good drug target (18), and inhibitors of the mitochondrial enzymes dihydroorotate dehydrogenase (pfDHODH) (19) and complex III subunit cytochrome B (PfCytB) (20) have been identified.

For years, it has been difficult to perform proteomics experiments to identify the *Plasmodium* mitochondrial proteome due to the lack of organelle isolation methods that reliably separate mitochondria from the apicoplast (21). The definition of mitochondrial functions relied heavily upon homology studies (7, 22) and microscopic examination of individual proteins (Table S1). With the use of two biotin tagging approaches, 422 mitochondrial matrix proteins were identified in Toxoplasma gondii, a species related to *Plasmodium* that also has an apicoplast and a mitochondrion (16). Besides these mitochondrion-targeting approaches, hyperLOPIT (hyperplexed localization of organelle proteins by isotope tagging), a whole-cell biochemical fractionation technique, defined the subcellular localization of the T. gondii proteome. This provided, among others, a set of 220 soluble and 168 membrane-bound T. gondii mitochondrial proteins (23). Alongside these proteomic data, there is a wealth of other data sources providing information on mitochondrial localization in P. falciparum. The challenge remains to use and combine these heterogeneous data sets to reliably predict the mitochondrial proteome.

10.1128/mSphere.00614-21.1 TABLE S1 Gold standards, alternative sets, and prediction results. Download Table S1, XLSX file, 2.8 MB. Copyright © 2021 van Esveld et al. 2021 van Esveld et al. https://creativecommons.org/licenses/by/4.0/ This content is distributed under the terms of the Creative Commons Attribution 4.0 International license.

Here, we integrated eight data sets containing proteomic data, gene expression data, data on orthology to mitochondrial proteins from other species, phylogenetic distribution data, and amino acid sequence data in a Bayesian manner, as has been applied before, such as for the definition of the mitochondrial proteome of humans (13) and of developmental stage-specific proteins in *Plasmodium* (24, 25). We used a naive Bayesian classifier to combine the data. This method exploits the relative strengths of the various data sets while maintaining transparency about the contribution of each data set to the final Bayesian posterior probability score for being mitochondrial. For each protein, the contribution of each data set to the prediction was determined using gold standards of *Plasmodium* proteins that are known to be either mitochondrial or nonmitochondrial. After data integration, we obtained a list of 445 P. falciparum mitochondrial proteins. We experimentally validated these predictions with seven proteins with various probability scores, and collectively, this predicted proteome indicates that the P. falciparum mitochondria are unique relative to mitochondria of model organisms.

## RESULTS

**FIG 1:**
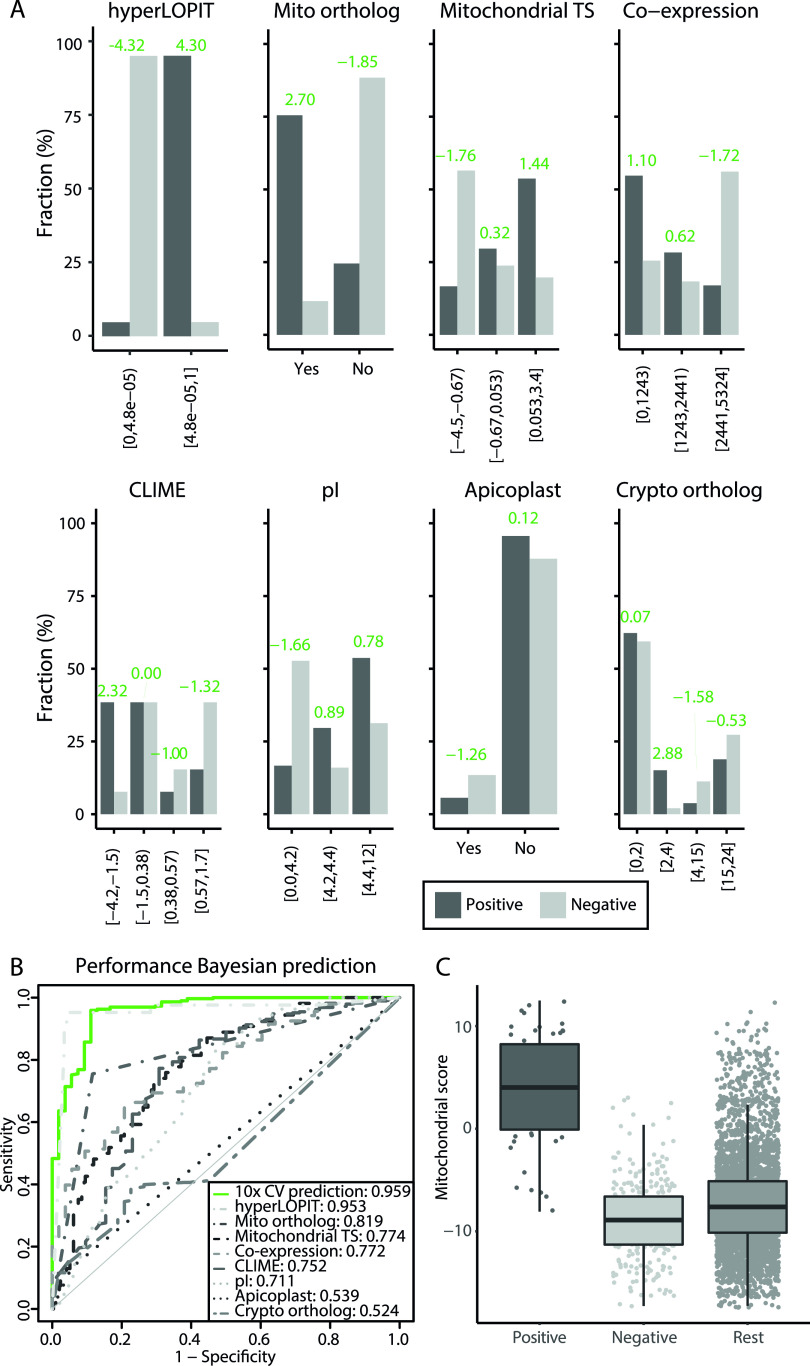
Predictive values of the individual data sets and the integrated ranked list. (A) Bar plot of each data set with the fraction of the gold standards on the *y* axis and the bins on the *x* axis. The mitochondrial score per bin (green) is given to show the predictive value of each bin. (B) ROC-curves comparing the performance to identify mitochondrial proteins of the prediction (10-fold cross-validated) to the performance of the individual data sets. For comparison, the values for the area under each curve are given in the legend. (C) Box plot that visualizes the distribution of the gold standard positives, negatives, and remainder of the proteome (*x* axis) over the calculated mitochondrial score of the prediction (*y* axis).

**FIG 2:**
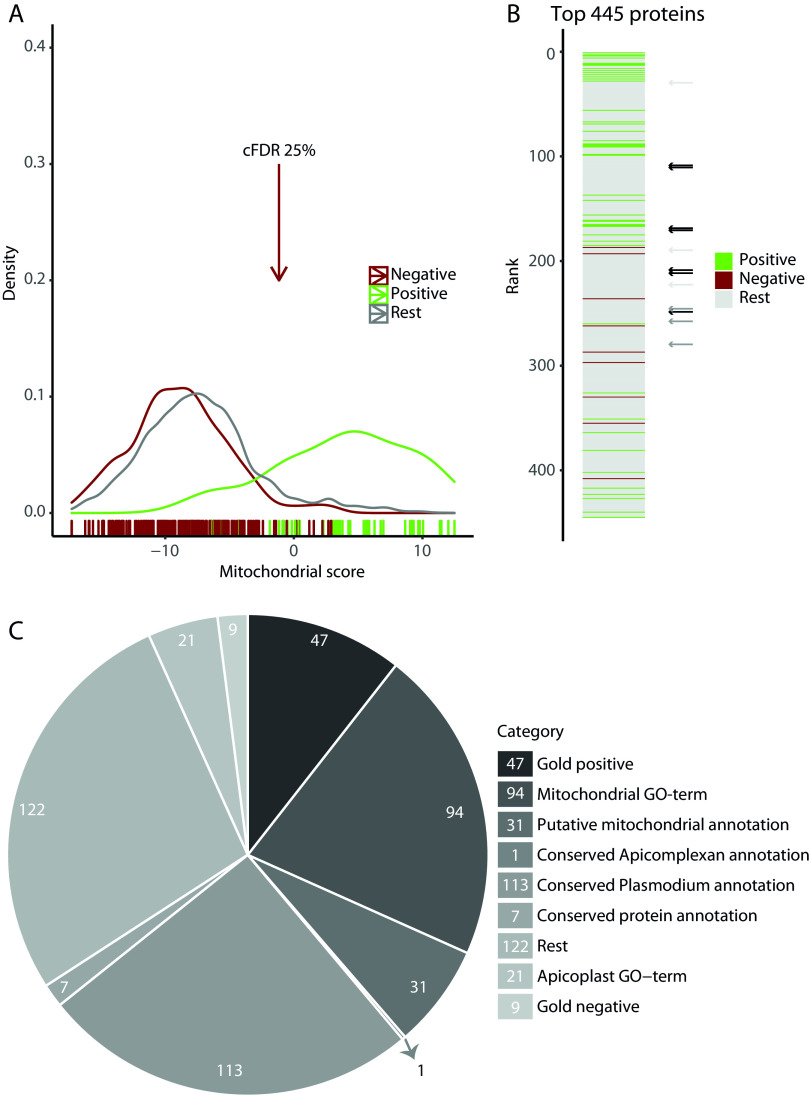
Integration produces a ranked proteome with 445 likely mitochondrial proteins at a 25% cFDR cutoff. (A) Density plot of the mitochondrial score with a colored bar at the bottom indicating the scores of individual gold standards. The arrow indicates the 25% cFDR cutoff. (B) The top 445 proteins falling in the 25% cFDR cutoff were ranked on their mitochondrial score, with color indicating if the protein is part of a gold standard. Black arrows indicate the rankings of the seven candidate proteins that we experimentally confirmed to be mitochondrial (Fig. 3A and B), dark gray arrows indicate the rankings of the three proteins with suggested mitochondrial localization (Fig. 3C), and light gray arrows indicate the rankings of the three proteins with inconclusive results (Fig. S4B). (C) Categorical representation of the 445 proteins identified at the 25% cFDR cutoff. First, genes were separated into categories based on the gold standard; the remainder were separated based on mitochondrial and apicoplast GO terms, and subsequently, the remainder were separated based on gene function annotations.

We integrated eight features of proteomic, gene expression, orthology, and amino acid composition data to predict mitochondrial localization in P. falciparum (Table 1) (Materials and Methods). All the individual features had a predictive value for mitochondrial localization (Fig. 1A and B), including, as expected, a negative score for the “*Cryptosporidium* ortholog” and the “apicoplast” data sets (Fig. S2A). Note that for the coexpression feature, it is the bin with the low-rank values that contains the most mitochondrial proteins, because this bin contains the proteins with the highest coexpression values. The best-performing input data sets are the ones that translate mitochondrial localization between species: the hyperLOPIT data set and the mitochondrial ortholog data set, followed by the mitochondrial targeting signal and coexpression data sets (Fig. 1B). Correlation analyses showed that the features are largely independent of each other, allowing us to use naive Bayesian data integration (Fig. S2B). The highest correlations were observed between features that examine the phylogenetic distribution of the proteins: CLIME (clustered by inferred models of evolution) and *Cryptosporidium* ortholog data sets. The log-odds ratios resulting from the Bayesian data integration achieve an area under the curve (AUC) of 0.959, indicating a very robust separation of mitochondrial and nonmitochondrial proteins (Fig. 1B). By integrating all data, we achieved both a better sensitivity and specificity in predicting mitochondrial proteins than we achieved for individual data sets. The performance of the hyperLOPIT data set on its own approximates the integrated prediction with an AUC of 0.953 (Fig. 1B). However, this data set contains information on only 2,246 (42%) of the nucleus-encoded *Plasmodium* proteins, highlighting the value of additional data sets and their integration to predict a complete mitochondrial proteome. The distribution of the log-odds ratios (Fig. 1C) showed that the mitochondrial score separated the positive and negative gold standards well from each other and that there are additional proteins with a high score that were not part of either, which are potential new mitochondrial proteins. We used a corrected false discovery rate (cFDR) of 25% (Fig. 2A) as a threshold for highly probable new mitochondrial proteins and identified 445 proteins (Fig. 2B) with a sensitivity of 87% and a specificity of 97%. The ranked list of the complete nucleus-encoded proteome is available in Table S1.

10.1128/mSphere.00614-21.5 FIG S2 Features of the input data sets. (A) Bar plots that visualize the fraction of apicoplast proteins, mitochondrial proteins, dually localized mitochondrial and apicoplast proteins, and the remainder of the proteome per bin for the data sets Apicoplast and *Cryptosporidium* ortholog. In this figure localization categories were based on mitochondrial and apicoplast associated GO terms and not on gold or alternative standards. (B) Heat map depicting the pairwise Spearman correlation coefficients between the input data sets used to predict the mitochondrial proteome. Download FIG S2, EPS file, 0.8 MB. Copyright © 2021 van Esveld et al. 2021 van Esveld et al. https://creativecommons.org/licenses/by/4.0/ This content is distributed under the terms of the Creative Commons Attribution 4.0 International license.

10.1128/mSphere.00614-21.7 FIG S4 Localization of selected predicted mitochondrial proteins in Plasmodium berghei. Tagged proteins of interest (POI) were stained with anti-HA antibodies (red), and the corresponding mitochondrial GFP markers were stained with anti-GFP antibodies (green). DNA was stained with DAPI (blue). (A) Representative images of candidate proteins tagged with an mOrange-3×HA tag, imaged on a Leica SP8 confocal microscope. (B) Representative images of candidate proteins tagged with a linker-3×HA tag, imaged on a Zeiss LSM900 confocal microscope. The histogram depicts normalized pixel intensities of the POI and mitochondrial marker plotted over the line indicated in the merge panel. Bars, 2 μm. Download FIG S4, EPS file, 2.7 MB. Copyright © 2021 van Esveld et al. 2021 van Esveld et al. https://creativecommons.org/licenses/by/4.0/ This content is distributed under the terms of the Creative Commons Attribution 4.0 International license.

### Comparison of the *Toxoplasma* and *Plasmodium* mitochondrial proteomes.

Overall, the hyperLOPIT data set contains 388 T. gondii mitochondrial proteins with a TAGM-MCMC location probability of 99% or higher. Of those, 296 have a P. falciparum ortholog, and of these orthologs, we found 273 in our predicted mitochondrial proteome. Most proteins that we predicted to be mitochondrial that were not supported by the T. gondii data are proteins that do have orthologs in T. gondii but for which there were no hyperLOPIT data (95 proteins). There are, furthermore, 38 proteins in the set of 445 that do not have T. gondii orthologs. It should be noted that these do not include the six P. falciparum mitochondrial ribosomal proteins that, based on BLAST searches, were deemed to be absent from T. gondii (26), as for those we could identify orthologs using HHpred (Table S1), underlining the relevance of sensitive homology detection. Finally, there are 39 proteins whose orthologs are present in the hyperLOPIT data set but which fell outside the 99% cutoff for mitochondrial localization that was used in that study; 26 of these did still have a mitochondrial localization as the most probable location, and 13 had a different predicted location in T. gondii (flagged in Table S1). Manual examination of those 13 inconsistencies revealed proteins like PF3D7_1125300/mitochondrial RNA polymerase, which in the hyperLOPIT data has been observed in the nucleolus, but also PF3D7_1446400/pdhB, which was predicted to be mitochondrial based on orthology with pdhB in, e.g., Homo sapiens but is localized in the apicoplast in *Plasmodium* (27).

### Validation of candidates.

**FIG 3:**
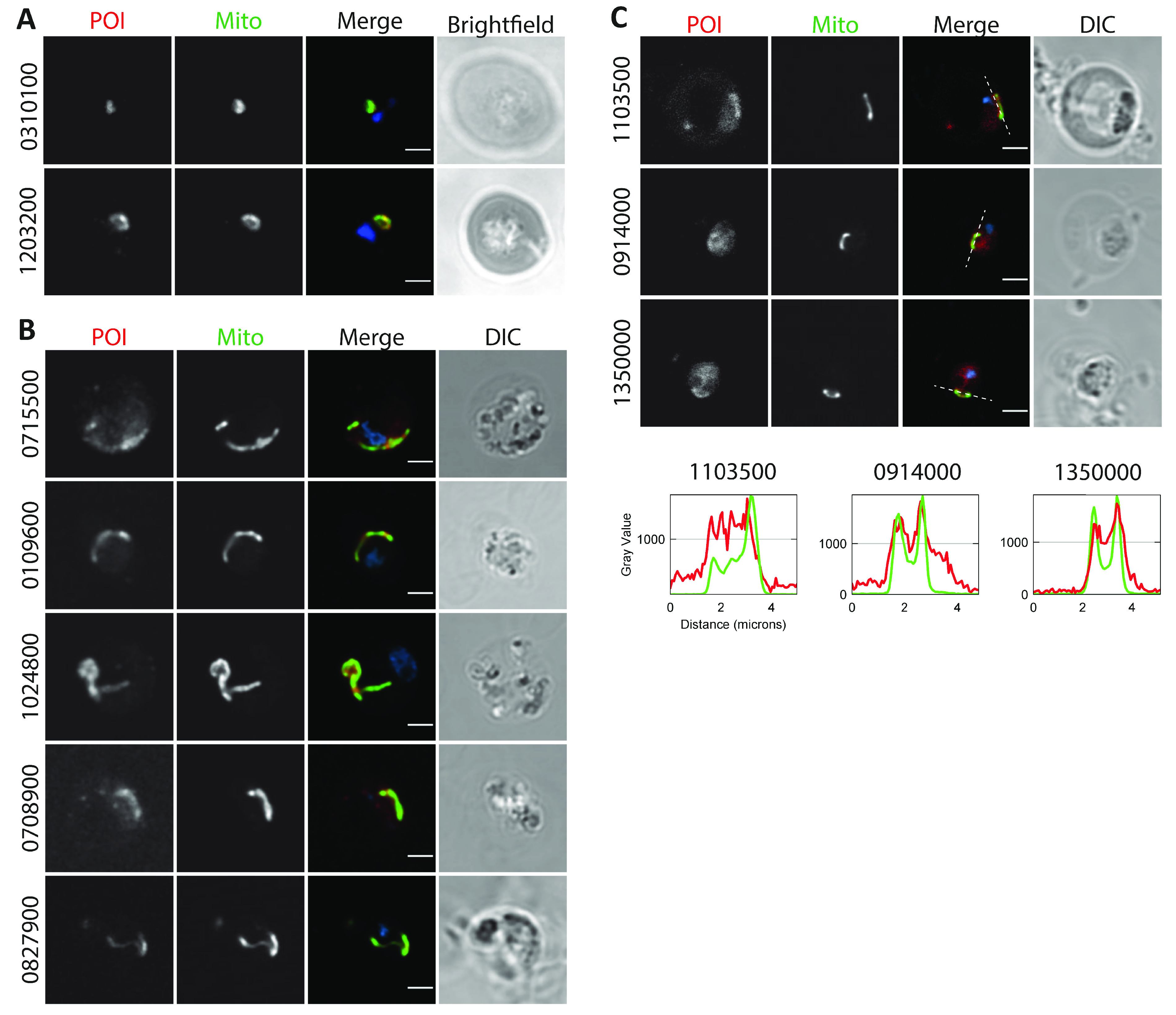
Validation of mitochondrial candidate proteins. Immunofluorescent analysis of tagged candidate proteins of interest (POI) in P. berghei parasites (PBANKA IDs are indicated on the left). POI were stained with anti-HA antibody (red; first columns), mitochondria were stained with anti-GFP antibody binding to the organelle marker (Mito; green; second columns). The DNA was stained with DAPI (blue; merge; third columns). (A) Representative images of candidate proteins tagged with an mOrange-3×HA tag, imaged on a SP8 confocal microscope (Leica). (B and C) Representative images of candidate proteins tagged with a linker-3×HA tag, imaged on an LSM900 confocal microscope (Zeiss). (C) Images with the brightest signal for three candidate proteins with low expression. Pixel intensities of the POI and mitochondrial signal were normalized and plotted over the line indicated in the merge panel. Bar, 2 μm.

We aimed to validate the mitochondrial candidate list by tagging orthologs of the selected unusual or unique predicted mitochondrial proteins in the efficient transfection model P. berghei (see “New mitochondrial proteins” for the P. falciparum orthologs of these proteins). Mitochondrial localization was assessed by fluorescence microscopy using an experimental genetic approach developed in our lab (17). This method allows the endogenous tagging of a target protein with a combined fluorescent and epitope tag, while simultaneously introducing a strong mitochondrion-targeting green fluorescent protein (GFP) marker by fusion of the promoter and N terminus of HSP70-3 (PBANKA_0914400) to GFP. Initially, we tagged 6 proteins with an mOrange-3×HA (hemagglutinin) tag. Live imaging revealed very poor and undefined signals (data not shown). Next, we fixed blood samples and stained them with anti-HA antibodies. Using this approach, only PBANKA_0310100 and PBANKA_1203200 were convincingly shown to localize to the mitochondrion (Fig. 3A), while other proteins demonstrated mostly undetectable or undefined signals (Fig. S4A). Since many of the selected proteins are relatively small compared to the mOrange tag and are expected to be imported into the mitochondrion, we anticipated that interference of the tag with import or proper folding could lead to the observed weak and undefined signals. To assess this, we selected two similarly small and abundantly expressed targets, PBANKA_0310100 (127 amino acids) and PBANKA_1024800 (144 amino acids), to test different tags. For PBANKA_0310100, which was already successfully localized to the mitochondrion, these included tags consisting of only the fluorescent protein mOrange or only the 3×HA epitope tag, either with or without a linker sequence. For PBANKA_1024800, we also included the combined mCherry-cMyc tag that has previously been used successfully to tag mitochondrial proteins (17). We found that the fluorescent protein tag indeed interfered with the localization of PBANKA_1024800, while the 3×HA tag, with or without a linker, supported a mitochondrial localization (Fig. S5B). Surprisingly, using the mOrange tag without the subsequent HA tag also led to an undefined rather punctuate mislocalization of PBANKA_0310100 (Fig. S5B). Based on these observations, we decided to tag the remaining 11 proteins with a linker-3×HA tag (Fig. 3B and C and Fig. S4B). Five of these were also convincingly localized to the mitochondria (Fig. 3B), while an additional three proteins showed very weak staining patterns suggestive of a possible mitochondrial localization (Fig. 3C). The signals of the remaining three tagged proteins were unable to be distinguished from the background, and therefore, no subcellular localization could be assigned (Fig. S4B).

10.1128/mSphere.00614-21.8 FIG S5 Effects of different tags on protein localization. (A) PBANKA_0310100 and (B) PBANKA_1024800. The tag integrated in the target locus is depicted vertically in black and the visualized signal of the protein of interest in gray. mOrange and mCherry signals (red) and the corresponding mitochondrial GFP markers (green) were visualized after fixation without the use of immunofluorescence. The 3×HA tags were stained with anti-HA antibodies (red) and corresponding GFP markers were stained with anti-GFP antibodies (green). DNA was stained with DAPI (blue). Bars, 2 μm. For completeness, the image of PBANKA_0310100 (visible in Fig. 3A) is repeated in this figure. Download FIG S5, EPS file, 2.2 MB. Copyright © 2021 van Esveld et al. 2021 van Esveld et al. https://creativecommons.org/licenses/by/4.0/ This content is distributed under the terms of the Creative Commons Attribution 4.0 International license.

### New mitochondrial proteins.

A categorical representation of the identified proteins (Fig. 2C) shows that 172 of the 445 proteins have a previous annotation of being mitochondrial, as they either are part of the gold standard, have a mitochondrial gene ontology (GO) term, or are described as putative mitochondrial proteins. A smaller number of proteins, 30, were part of the negative set or have an apicoplast GO term and no mitochondrial GO term. The remaining 243 proteins, of which 121 are annotated as conserved hypothetical proteins, contain new mitochondrial candidates.

For 27 of the 121 conserved proteins with an unknown function, we found a mitochondrial ortholog using the sensitive homology detection tool HHpred (28), which can improve their annotation (Table S1). For example, the human and yeast orthologs of PF3D7_0306000 (Bayesian rank 41) are both cytochrome *b*-*c1* complex subunit 8, making it very likely that the *Plasmodium* protein is also cytochrome *b*-*c1* complex subunit 8, as confirmed by recent complexome profiling (12). Other similar examples include PF3D7_1357600 (rank 285), which is likely a mitochondrial member of the 39S ribosomal protein family L53, and PF3D7_1309900 (rank 80), which is likely succinate dehydrogenase assembly factor 2.

Besides the possibility of improving annotations using well-characterized mitochondrial orthologs, the predicted 445 proteins contain biologically interesting proteins that can add new mitochondrial functions to P. falciparum and related species. Thus, the prediction provides targets for experimental research to uncover new biology. These proteins can be divided into five categories: (i) proteins with known mitochondrial orthologs and an unknown mitochondrial role in P. falciparum, (ii) proteins without a mitochondrial ortholog but with homology to proteins with a known function and conservation of critical residues that suggest conservation of that function, (iii) proteins with homology to proteins with a known function from which residues known to be essential for catalytic activity or for binding substrates residues have been lost, (iv) new proteins of a known mitochondrial protein family, and (v) proteins of a new mitochondrial protein family. Below, we describe several proteins per category, including their domain structure, the conservation of active site residues, their phylogenetic distribution, and, if available, the experimental confirmation of their mitochondrial localization.

### Proteins with known mitochondrial orthologs and an unknown mitochondrial role in P. falciparum.

PF3D7_0413500/PfPGM2 (rank 111) contains a PGM domain and appears to be orthologous to the human serine/threonine-protein phosphatase, PGAM5, of which the catalytic residue H105 (29) is conserved (30). PfPGM2 catalyzes the dephosphorylation of phosphorylated sugars and amino acids (30). In human cells, PGAM5 is localized to the mitochondrial outer membrane (31), where it plays a role in apoptosis, mitofission, and mitophagy (reviewed in reference 32), among others, via interactions with BCL2-like protein 1 (33). A previous study reported an unexpected cytoplasmic localization of this protein in P. falciparum and P. berghei, though in the absence of bright-field/differential interference contrast (DIC) images or colocalization with marker proteins, it is difficult to draw conclusions on the localization (30). Therefore, we tagged the P. berghei ortholog PBANKA_0715500 with a 3×HA tag and confirmed colocalization of PbPGM2 with our mitochondrial marker (Fig. 3B). We also observed a weak nonmitochondrial background signal, which could indicate a potential dual localization. An important consideration in the interpretation of the previously reported localization of the GFP-tagged P. berghei protein is our observation that the use of a big tag such as GFP can interfere with the localization of mitochondrial proteins (Fig. S5).

Originally, we flagged two additional proteins, PF3D7_0213200 (rank 169) and PF3D7_0611300 (rank 171), as interesting due to potential homology to BCL2, based on sequence similarity and a similar alpha-helical structure predicted with HHpred (28). PF3D7_0213200 was assigned an apicoplast GO term, but in our analysis, it is a potential mitochondrial protein. Analysis with TMHMM (34) indicates that it is probably a transmembrane protein with an in-out topology. The protein PF3D7_0611300 had already been assigned a mitochondrial GO term based on a screening in T. gondii (35) but was, to our knowledge, not confirmed as mitochondrion localized by experiments characterizing this protein in P. falciparum. Although we rejected BCL2 homology for both proteins after a detailed examination of the residues conserved in the BCL2 family, we confirmed the mitochondrial localization of PF3D7_0213200 (PBANKA_0310100 in Fig. 3A) and PF3D7_0611300 (PBANKA_0109600 in Fig. 3B). Indeed, complexome profiling revealed that the latter is part of the ATP synthase complex (12).

### Proteins with a nonmitochondrial homolog and conservation of critical residues.

PF3D7_0913400 (rank 30) is homologous to bacterial elongation factor P. Although eukaryotes contain initiation factor 5A as a cytoplasmic elongation factor P homolog, this protein family was until now not observed in mitochondria. PF3D7_0913400 contains both the N-terminal KOW-like domain and the central P/YeiP domain of elongation factor P, but not the C-terminal OB domain. Elongation factor P plays a role in translation elongation and specifically in the translation of stretches of 2 or more prolines (36). A basic residue at position 34 of elongation factor P (numbering from the Escherichia coli PDB structure 3A5Z_F [37]), which, after posttranslational modification, interacts with the CCA end of the tRNA, is conserved in PF3D7_0913400. Notably, mitochondrially encoded P. falciparum cytochrome *c* oxidase subunit 1 contains a proline pair at positions 135 and 136, making it a potential target for elongation factor P.

PF3D7_0812200 (rank 147) is homologous to the bacterial DegP protease family. It contains both a serine endoprotease domain and a PDZ domain. The DegP from E. coli functions in acid resistance in the periplasm. It is able to refold after acid stress and subsequently can cleave proteins that are misfolded due to acid stress (38). The active-site residue (S210 in PDB structure 1KY9 [39]) is conserved in PF3D7_0812200, but residues involved in allosteric loop interactions, including R187, are not. Orthologs of PF3D7_0812200 appear, among the eukaryotes, to be limited to the Apicomplexa and Chromerida.

PF3D7_0503900 (rank 246) has a C-terminal dioxygenase domain (E = 1.3E−16). The closest homolog in human is phytanoyl coenzyme A (phytanoyl-CoA) dioxygenase, which resides in the peroxisome, an organelle absent from *Plasmodium*, where it is involved in α-oxidation of branched-chain fatty acids. Important residues in the active site that are involved in iron binding, H175, H177, and H264 (40), are conserved in PF3D7_0503900, suggesting that it is a functional enzyme. Nevertheless, levels of sequence conservation are low (14% identity with the human phytanoyl-CoA dioxygenase), and we did not detect conservation of substrate binding sites with the phytanoyl-CoA dioxygenase. The colocalization analysis suggested a mitochondrial localization for PF3D7_0503900 (PBANKA_1103500 in Fig. 3C).

PF3D7_1121500 (rank 307) contains a papain-like NLpC/P60 superfamily domain (Peptidase_C92 in PFAM). This protein family contains, among others, cysteine peptidases and amidases. Of the four residues essential for activity in BcPPNE from Bacillus cereus (41), the most similar experimentally characterized homolog of PF3D7_1121500, three are conserved (H49, E64, and Y164).

### Proteins with homology to proteins with a known function from which residues known to be essential for catalytic activity or for binding substrates residues have been lost.

PF3D7_1004900 (rank 212) is homologous to the protein component of the signal recognition particle (SRP), a ribonucleoprotein that recognizes and targets specific proteins to the endoplasmic reticulum (ER) in eukaryotes. PF3D7_1004900 contains the C-terminal M domain (E = 5.8E−25), but not the other domains, SRP54 and SRP54_N domains, of this protein. In the SRP, the M domain binds both the SRP RNA and the signal sequence of the target protein. Inspection of individual amino acids did not reveal conservation of RNA-binding amino acids (e.g., the arginines in helix M4 of the M domain [42] are not conserved) but did reveal some conservation of hydrophobic amino acids lining the groove in which the signal peptide is located. These hydrophobic amino acids have been implicated in interactions with the signal peptide, specifically, V323, I326, and L329 in the M1 helix and L418 in the M5 helix (positions relative to Sulfolobus solfataricus structure 3KL4 [42, 43]), suggesting a possible conservation of interaction of PF3D7_1004900 with a hydrophobic α-helix. We experimentally confirmed the mitochondrial location of PF3D7_1004900 (PBANKA_1203200 in Fig. 3A).

PF3D7_1246700 (rank 223) is a member of the pyridoxamine 5′-phosphate oxidase (PNPOx)-like protein family, which, besides pyridoxamine 5′-phosphate oxidase, also contains the general stress protein 26 family. The most similar experimentally characterized protein is general stress protein 26 from Xanthomonas citri, encoded by the gene *Xac2369*. The latter binds flavin adenine dinucleotide (FAD) and flavin mononucleotide (FMN) but not pyridoxal 5′-phosphate, indicating that it does not function as a PNPOx (44). Relative to the unpublished structure of a protein from this family from Nostoc punctiforme (PDB code 2I02), which does contain an FMN, we did not observe conservation of the amino acids, like W111, that line the FMN binding pocket, casting doubt on a function for PF3D7_1246700 that includes the binding of FMN.

### New members of mitochondrial protein families.

PF3D7_1417900 (rank 109), PF3D7_1142800 (rank 172), and PF3D7_0927100 (rank 249) potentially contain a CHCH domain. They all have two pairs of cysteines that are very well conserved among their detectable homologs, and the nine amino acids between the cysteines are predicted to form an α-helix (28). Nevertheless, as nothing other than cysteines appears to be conserved, the E value with the PFAM CHCH domain entry, or with any known mitochondrial protein, is not significant (E > 0.01). Jackhmmer analysis (45) detects homologs among the Apicomplexa, including a homolog in *Cryptosporidium muris*, but not outside this taxon. Known proteins with a CHCH domain are localized to the mitochondrial intermembrane space, where they can participate in disulfide bond formation, suggesting that PF3D7_1417900, PF3D7_1142800, and PF3D7_0927100 might also be in the intermembrane space. Complexome profiling revealed that the first two are part of the ATP synthase complex (12). We tested PF3D7_1417900 and PF3D7_0927100 experimentally and confirmed them to be mitochondrial (PBANKA_1024800 and PBANKA_0827900 in Fig. 3B, respectively). Thus, based on our data and the complexome profiling data, all three proteins are confirmed to be mitochondrial, increasing the likelihood that they indeed contain a CHCH domain.

*Plasmodium* contains a cytochrome *c* heme lyase (PF3D7_1224600; rank 129) and a cytochrome *c*_1_ heme lyase (PF3D7_1203600; rank 225), which are both essential during blood-stage development and have nonoverlapping functions (46). We uncovered a third homolog in this family at rank 190, PF3D7_1121200 (E = 0.0096; HHpred [28]). Although the homology covers domains II, III, and IV of heme lyases, of which domain II has been implicated in binding heme (47), residues that have been implicated in heme binding, like H154 (coordinates for human protein [47]) and residues of domain I are not conserved in PF3D7_1121200. The protein also occurs in ciliates and dinoflagellates but has no orthologs outside those taxa and thus appears to be an alveolate-specific duplication.

### A new apicomplexan mitochondrial protein family.

We assessed to what extent *Plasmodium* mitochondrial proteins were part of families with multiple mitochondrial members that resulted from duplications (Table S1). Such “intracompartmental protein duplications” have also increased the size of the human mitochondrial proteome (48). Among the mitochondrial homologs, we uncovered a new family in the Apicomplexa that in *Plasmodium* species has four representatives: PF3D7_0821900 (rank 209), PF3D7_1336100 (rank 258), PF3D7_1134400 (rank 280), and PF3D7_1228000 (rank 499). The family is characterized by a well-conserved WPP motif at its N terminus (Fig. S7), and we therefore name it the WPP family. Although PF3D7_1228000 does not rank sufficiently high to fall within the 25% cFDR cutoff, all proteins of the family are likely mitochondrial given their overall high ranking and the tendency of members of protein families to be localized in the same cellular location. For the protein family itself, we did not detect homologs outside the Apicomplexa and *Vitrella*. In several species, including Plasmodium coatneyi (PCOAH_00054040), Babesia bovis (BBOV_III010100), and Theileria orientalis (MACL_00002472), the ortholog of PF3D7_1228000 is predicted to be fused with an rRNA pseudouridylate synthase, hinting at a potential function of this family in rRNA maturation. Nevertheless, there are no experimental data supporting those gene fusions. We unequivocally confirmed the mitochondrial location of PF3D7_0821900 (PBANKA_0708900 in Fig. 3B), and the colocalization analysis also suggested a mitochondrial localization for PF3D7_1336100 and PF3D7_1134400 (PBANKA_1350000 and PBANKA_0914000 in Fig. 3C, respectively). PF3D7_1228000 was excluded from the analysis due to low transcript levels.

10.1128/mSphere.00614-21.9 FIG S6 Diagnostic PCRs of Plasmodium berghei transfection experiments. Integration PCR results on genomic DNA from parasites before (left) and after (right) transfection using the primer combinations shown in Fig. S3 and Table S4. Vertical numbers refer to the respective PBANKA IDs. (A) Assessment of integration of the mOrange-3×HA tag. (B) Integration of multiple tags for PBANKA_0310100 and PBANKA_1024800. mOr, mOrange; HA, 3×HA; mCh, mCherry. (C) Assessment of integration of linker-3×HA tag. Download FIG S6, PDF file, 2.0 MB. Copyright © 2021 van Esveld et al. 2021 van Esveld et al. https://creativecommons.org/licenses/by/4.0/ This content is distributed under the terms of the Creative Commons Attribution 4.0 International license.

10.1128/mSphere.00614-21.6 FIG S3 Schematic overview of tagging strategy. For all transfections, the same double-crossover homologous recombination strategy was applied, resulting in endogenously and stably tagging of the gene of interest (GOI). Every transfection vector contained a drug-selectable and a mitochondrial GFP marker cassette and was integrated into the genome via the C terminus (CT) and 3′ untranslated region (UTR) (3′). As a result, the proteins of interest are fused in frame to the various tags applied in this study as indicated in the text, allowing colocalization studies with the mitochondrial GFP marker. Primers used for the diagnostic PCR specific for wild-type (WT) or successful integration (INT) are indicated (a to d) and correspond to those listed in Table S2 FIG S3, EPS file, 0.4 MB. Copyright © 2021 van Esveld et al. 2021 van Esveld et al. https://creativecommons.org/licenses/by/4.0/ This content is distributed under the terms of the Creative Commons Attribution 4.0 International license.

10.1128/mSphere.00614-21.10 FIG S7 Sequence logo of the mitochondrial WPP family. An alignment was made using Jackhmmer (45) on the RefSeq database, starting with PF3D7_0821900 and iterating until convergence. The alignment was subsequently pasted in the sequence logo tool https://weblogo.berkeley.edu/ to obtain a graphical representation of the levels of conservation. Download FIG S7, EPS file, 0.7 MB. Copyright © 2021 van Esveld et al. 2021 van Esveld et al. https://creativecommons.org/licenses/by/4.0/ This content is distributed under the terms of the Creative Commons Attribution 4.0 International license.

## DISCUSSION

In this work, we ranked all nucleus-encoded proteins of P. falciparum for their likelihood of being mitochondrially targeted and defined the top 445 proteins (cFDR, 25%) as likely mitochondrial proteins (Table S1). Despite the large variation between the data sets in the coverage of the nuclear encoded proteome and in the ability to identify mitochondrial proteins (Table 1; Fig. 1), the probabilistic approach we used was able to integrate the data in an unbiased way by assessing the distribution of gold standard genes within each data set during the scoring process.

**TABLE 1:** Input data sets for Bayesian integration

Dataset	Abbreviation used in figures	Size of data set	Fraction identified (%)	Fraction gold standard identified (%)	*P*
Data set based on omics data					
Coexpression, built using in-house developed WICCA tool (rank ≤ 2400) (https://wicca.cmbi.umcn.nl/)	Coexpression	5,310	45.6	83.0	2.0E−08

Non-apicoplast proteins (58)	Apicoplast	5,324	93.6	94.4	5.4E−01

Data sets based on ortholog evidence					
hyperLOPIT (TAGM.MCMC.joint.mitochondrion_max ≥ 4.78E-05) (23)	hyperLOPIT	2,246	15	95.2	9.3E−32
Mitochondrial ortholog	Mito ortholog	5,280	8.7	75.5	2.6E−32
Evolutionary inference, built using CLIME (NN score ≤ −1.5) (63)	CLIME	4,687	9.0	38.5	6.9E−09
Absence of a *Cryptosporidium* ortholog (score ≤ 4)	Crypto ortholog	5,280	67.5	77.4	7.9E−02

Data sets based on amino acid sequence evidence					
Mitochondrial targeting signal, built using PFMpred tool (SVM ≥ −0.67) (67)	Mitochondrial TS	5,324	59.5	83.3	1.4E−04
Isoelectric point (Patrickios pI ≥ 4.2) (70)	pI	5,324	55.1	83.3	1.1E−05

### Quality of the predictions.

Several observations indicate the high quality of the predictions. First, the 10-fold cross-validation with an area under the curve of 0.959 shows the high predictive value and robustness of the prediction. Second, almost half of the predicted proteins have a previous annotation of being mitochondrial, even though most of these were not used in weighing the data sets. Third, of 13 proteins that we set out to validate experimentally, the seven for which we obtained a high enough expression to convincingly locate them in the cell were all mitochondrial, while for three others, the experimental data were consistent with a mitochondrial localization but equivocal due to low expression levels. Fourth, even though our top 445 contain nine proteins of the negative gold standard that was not manually supervised for all entries, manual examination of those nine showed that the majority are likely mitochondrial after all. Seven of them were detected in the hyperLOPIT study (23), and of those, five were annotated as mitochondrial (see the legend to Table S1). Finally, several proteins that we predicted to be mitochondrial have, since we made those predictions, been shown to be mitochondrial using other methods. Specifically, complexome profiling of mitochondrion-enriched fractions has elucidated the composition of mitochondrial complexes II, III, IV, and V (12). We identified in our top 445 proteins six of the seven members of complex II (four of which are not in the Gold Standard (GS) or mitochondrial in Gene Ontology [GO]), 10 of the 11 members of complex III (none in the GS and three not in GO), 17 of the 19 members of complex IV (none in the GS and 13 not in GO), and 21 of the 23 members of complex V (15 not in the GS and nine not in GO) (Table S1). These results reinforce the robustness of the predictions and confirm that the ranked list of the complete nucleus-encoded P. falciparum proteome, which we named PlasmoMitoCarta, is a useful resource for *Plasmodium* researchers.

### New mitochondrial biology.

The results of the integration provide ample material for exploring new mitochondrial biology, as 185 out of the 445 proteins do not have a detectable mitochondrial ortholog in yeasts, humans, or plants. Some, however, do have homologs in bacteria that include conservation of critical residues, for example, elongation factor P, whose function, if confirmed, would be unique to mitochondria. They also include paralogs of the known mitochondrial proteins, like the twin Cx9C and heme lyase families, and a new mitochondrial protein family that contains a well-conserved WPP motif. Finally, they include PfPGM2, a protein which does have a human mitochondrial ortholog but for which the experimental data thus far had pointed to a cytoplasmic organization (30). The integrated omics data and localization with a 3×HA tag do point at a mainly mitochondrial localization for a protein that in human is involved in apoptosis, mitofission, and mitophagy, processes about which little is known in *Plasmodium.* Such proteins provide leads to new mitochondrial biology.

As part of this study, we provide two other valuable resources. First, we included the ortholog information gathered for this study, which can improve the annotation of P. falciparum proteins and provide researchers with additional hints for the function of a protein (Table S1). In addition, the WICCA tool that we developed for this study is available online. It allows any researcher to assess coexpression of P. falciparum genes with a set of genes of four *Plasmodium* species across data from 83 microarray expression experiments, by weighing the data sets for their relevance to the set of genes. This can aid in the discovery of novel members of known pathways and molecular systems.

### Size of the mitochondrial proteome.

We predict 445 mitochondrial proteins at the cutoff, set at a cFDR of 25%. When using the posterior probabilities (2^mitochondrial score^) of all P. falciparum genes to calculate the estimated number (E) of mitochondrial proteins by summing them all up {E = sum[*P*_posterior_/(*P*_posterior_ + 1)]} (see reference 49 for a detailed explanation), we predicted the total size of the mitochondrial proteome to be 454 proteins. It therefore would appear that our prior odds of ∼10%, which affects the total size calculation, overestimated the number of mitochondrial proteins. One can in principle lower the prior odds (49) to 8.3% (412 proteins), such that the number is consistent with the estimated size of the proteome based on all data. However, the presence of five likely mitochondrial proteins in our gold negative sets leads to an underestimate of the size of the mitochondrial proteome. We decided not to manually weed out these inconsistencies, as this cannot be done systematically for all data sets, and applying this only to the apparent inconsistencies would result in circular arguments.

The predicted total size of 454 proteins is relatively small compared to the mitochondrial proteomes in humans, plants, and yeasts, which consist of at least 900 proteins (5, 50, 51). However, small mitochondrial proteomes have been reported before, for example, 573 proteins in the distantly related ciliate Tetrahymena thermophila (52). In agreement with our findings, a recent study on the mitochondrial proteome of T. gondii that was not included in our integration reports a proteome of 421 proteins (16).

In conclusion, we combined the information of eight data sets with the curated gold standards to rank all nucleus-encoded P. falciparum proteins on their likelihood of being mitochondrial in PlasmoMitoCarta. The value of this ranked proteome is shown by *in vivo* validation of top-scoring proteins in the closely related species P. berghei and will provide an important resource for future investigations.

## MATERIALS AND METHODS

### Animal experiments.

All animal experiments were performed in accordance with the Dutch Experiments on Animals Act (Wod) and Directive 2010/63/EU from the European Union and the European ETS 123 convention and were approved by the Radboud University Animal Welfare Body (IvD) and Animal Experiment Committee (RUDEC; 2015-0142) and the Central Authority for Scientific Procedures on Animals (CCD; AVD103002016424). In this study, we used outbred male and female NMRI mice (Envigo).

### Nucleus-encoded reference proteome.

All data sets used in this study were mapped to the P. falciparum 3D7 reference proteome, version 3.1, from the Sanger Institute, downloaded December 2017. This version, including isoforms, contains 5,431 proteins encoded by 5,357 genes. As we are interested in the proteins encoded by nuclear genes, all apicoplast and mitochondrion-encoded proteins were excluded, leaving a reference of 5,324 genes. When a data set contained informational data points for two or more protein isoforms of one gene, the value chosen for that gene was the one that came closest to the expected values for mitochondrial proteins in that specific data set.

### Assembly of positive and negative lists for benchmarking.

Bayesian data integration depends on gold standards of proteins known to be mitochondrial or nonmitochondrial. We constructed two gold standards to assess the predictive value of the individual data sets. Furthermore, we built two alternative sets for the construction of the CLIME (coevolution) and WICCA (coexpression) data sets to prevent a situation in which the standards chosen to train those two predictors were also used to evaluate them. Thus, we avoided circular arguments in the data integration. These four sets are unique, have no overlapping genes, and are available in Table S1. To construct these lists, we commenced with a systematic review of all literature available via PubMed (as of 30 August 2018) using the following broad search string: (mitochondri* OR apicoplast OR plastid) AND (plasmodium OR malaria). Given the intimate functional and physical relation between mitochondrion and apicoplast, we reasoned that the positive gold standard list should only include genes encoding proteins that had been unambiguously and singularly assigned to the mitochondrion through fluorescence microscopy and colocalization with confirmed mitochondrial markers or via immunoelectron microscopy (54 genes). Any proteins for which multiple studies showed different results were excluded. To facilitate discrimination from the apicoplast, we also made a gold standard apicoplast list (72 genes). Finally, all proteins that were unambiguously nonmitochondrial as demonstrated in the same papers (170 genes) were combined with the apicoplast-positive list to form the basis for the negative gold standard. This negative set was then complemented with an additional 346 nonoverlapping genes encoding nonapicoplast and nonmitochondrial proteins identified through extensive literature review and published online by the Ralph lab (53). The 588 genes encoding nonmitochondrial proteins were randomly assigned to the negative gold standard (294 genes) that was used for the Bayesian data integration and the alternative negative gold standard (294 genes) that was used to train the CLIME and WICCA approaches. For the latter purpose, we compiled an alternative positive standard of 146 genes, nonoverlapping with the first gold standard but associated with one or more mitochondrial GO terms (GO:0000275, GO:0005739-43, GO:0005746, GO:0005750, GO:0005753, GO:0005758, GO:0005759, GO:0006122, GO:0006839, GO:0006850, GO:0031966, GO:0033108, GO:0042775, GO:0044429, and GO:0044455).

### Data sets to predict mitochondrial and nonmitochondrial proteins.

To predict mitochondrial proteins and nonmitochondrial proteins, we collected and constructed eight data sets (Table 1) that contain features typical of either mitochondrial or apicoplast proteins. Some proteins, like lipoate protein ligase 2 (PF3D7_0923600) (54) and serine hydroxymethyltransferase isoforms (PF3D7_1235600) (55), are dually localized to the mitochondrion and apicoplast, highlighting the need for the second category of data sets. Each data set is described in detail below, including how the data set was processed to obtain for each P. falciparum protein a single score relevant to whether the protein is mitochondrial.

The size of data sets indicates the number of *Plasmodium* genes that could potentially have been measured in this data set. Fractions indicate the actually identified genes of this set that fall in bins with a positive mitochondrial score (Fig. 1A). *P* values (Fisher's exact test) indicate overrepresentation of identified gold standard genes compared to random expectation. Note that in “non-Apicoplast proteins” and in “Absence of a Cryptosporidium ortholog,” these *P* values reflect the overrepresentation of mitochondrial proteins among the large sets of proteins to which these features apply.

### Data set based on omics data. (i) Coexpression.

Coexpressed genes tend to code for proteins that functionally interact. For mammals, coexpression analyses have been successfully used in the discovery of mitochondrial proteins (5, 56). To determine if the expression pattern of a *Plasmodium* gene correlates with mitochondrial protein-coding genes, we developed a coexpression tool: Weighted Co-expression Calculation Tool for *Plasmodium* Genes (WICCA [https://wicca.cmbi.umcn.nl/]). In short, the tool uses the coexpression of a group of input genes with each other to weigh 83 microarray experiments for their predictive value for that group. Data sets that show high coexpression of the input genes are more likely to be relevant for the system that the input genes are part of and receive a higher weight. WICCA then uses these weights to combine the expression data sets and calculate one coexpression score per gene with the input system, in this case, one score per gene for the level of coexpression with mitochondrial-protein-coding genes. We used the coexpression ranking obtained with the alternative positive standard of 146 genes as input for WICCA to weigh the coexpression data sets. The methods of this tool were based on the WeGET method (http://weget.cmbi.umcn.nl/) (57); methodological details on WICCA can be found in the supplemental material.

### (ii) Bio-ID.

In Bayesian data integration, “negative data sets” that, e.g., predict that a protein is nonmitochondrial can be as valuable as positive ones. We used the set of 346 apicoplast proteins that are based on BioID data and gene expression data analyzed with a neural network (58) to reduce apicoplast protein contamination among the top-scoring proteins.

### Data sets based on ortholog evidence. (i) hyperLOPIT.

Hyperplexed localization of organelle proteins by isotope tagging (hyperLOPIT) is a proteomics technique that analyzes protein distributions upon biochemical fractionation. It enables the identification of the subcellular localization of thousands of proteins (59, 60). Barylyuk et al. (23) used this technique on the apicomplexan T. gondii and, among others, classified proteins identified in all three hyperLOPIT experiments to be part of the mitochondrial soluble matrix and mitochondrial membrane. This classification was based on t-augmented Gaussian mixture models (TAGM) in combination with maximum *a posteriori* prediction (TAGM-MAP) and Markov chain Monte Carlo (TAGM-MCMC) methods (23). Marker proteins used for the classification were arbitrarily set to 1 in the class that they belong to and to 0 for all other classes. Using BLAST (61) or, if that produced no homologs, HHpred (28), in combination with selecting for best bidirectional hits, we determined a list of orthologs between T. gondii and P. falciparum. The 3,832 T. gondii proteins that got a TAGM-MCMC probability for mitochondrial soluble matrix and mitochondrial membrane were mapped to the corresponding P. falciparum ortholog when available. This resulted in a list of 2,246 P. falciparum identifiers with two TAGM-MCMC probabilities, one for mitochondrial matrix localization and one for mitochondrial membrane localization. As the input data sets for the Bayesian integration should be independent, both TAGM-MCMC probabilities cannot be included as separate data sets. Therefore, the maximum TAGM-MCMC probability (either the soluble or the membrane value) for each protein was used to create one input hyperLOPIT data set.

### (ii) Mitochondrial ortholog.

We also used orthologs from more distantly related species than T. gondii, as at greater phylogenetic distances, subcellular localizations also tend to be conserved (48). Using BLAST (61) (all-versus-all BLAST for proteomes with an E value of 100) in combination with OrthoMCL (62) (percent match cut of 50; E value exponent cutoff of −5) to detect orthologs or, if that produced no result, HHpred (28) (default settings; E value cutoff of 0.01, three iterations, only best bidirectional hits included), one-to-one orthologs between P. falciparum and either Homo sapiens, Arabidopsis thaliana, or Saccharomyces cerevisiae were determined. When the orthologous protein of at least one species was annotated to be mitochondrial in a published mitochondrial compendium (H. sapiens, MitoCarta2.0 [5]; *A. thaliana* [50]; S. cerevisiae [51]), then that P. falciparum protein was included in the mitochondrial ortholog data set.

### (iii) Evolutionary inference.

Clustered by inferred models of evolution (CLIME) uses homology across model species (138 eukaryotic species and 1 prokaryotic outgroup) to identify evolutionary conserved clusters. We downloaded the precomputed CLIME analyses of P. falciparum genes from http://gene-clime.org/ (63) and mapped the IDs to our reference proteome. We used the CLIME matrix listing the presence/absence of orthologs of P. falciparum in other species, with the alternative positive and negative sets as training data, to train a perceptron with two hidden layers to obtain a single score per protein (details are presented in the supplemental material). The resulting output scores on our test set formed the CLIME input for the Bayesian integration.

### (iv) Absence of a *Cryptosporidium* ortholog.

Like *Plasmodium*, the genus *Cryptosporidium* belongs to the phylum Apicomplexa. *Cryptosporidium* species lack an apicoplast and contain only a remnant mitochondrion-like organelle (64). Therefore, it can be expected that apicoplast proteins and many mitochondrial proteins will not have an ortholog in *Cryptosporidium*. MetaPhOrs is an online tool that contains orthology and paralogy predictions obtained from multiple phylogenetic trees (65) and was used to assess orthology for P. falciparum genes in three *Cryptosporidium* species (Cryptosporidium hominis, Cryptosporidium parvum, and Cryptosporidium muris). We calculated a combined score by multiplying two MetaPhOrs metrics, one for the confidence level (see below) and one for the number of hits between the four species, and used this combined score as input for the Bayesian integration. In detail, the MetaPhOrs results include a consistency score (ranging from 0 with no overlap to 1 if all trees contain the same protein relationship/orthology information) and an evidence level for the number of independent databases (the theoretical maximum is 13, but for our species combination it was 4). The multiplication of consistency and evidence level results in an arbitrary score (range, 0 to 4) that indicates the confidence in calling two proteins orthologs. P. falciparum proteins with a high score are more likely to have an ortholog in *Cryptosporidium*. Notice that there is some overlap with the evolutionary inference, as that includes one *Cryptosporidium* species. Nevertheless, *Cryptosporidium* species show quite some variation in the complexity of their mitosomes, and the overlap in the predictions is limited (see Results).

### Data sets based on amino acid sequence evidence. (i) Mitochondrial targeting signal.

The canonical mitochondrial import system requires an amphipathic α-helical N-terminal targeting sequence. As *Plasmodium* has a distinct amino acid usage pattern (66), a P. falciparum-specific tool, PFMpred (67), was chosen to predict mitochondrial localization based on the amino acid sequence. This is a support vector machine (SVM) based tool and was used in split-amino-acid-composition mode (Matthews correlation coefficient, 0.73), which allows separate calculations for the N terminus, C terminus, and remainder of the protein. The tool reports one SVM score per protein and indicates whether the protein is predicted to be mitochondrial. The SVM scores per gene are used as the mitochondrial targeting signal data set.

### (ii) Isoelectric point.

Nucleus-encoded mitochondrial proteins need to cross the negatively charged mitochondrial membrane and need to function properly inside the mitochondrial environment, which has a slightly higher pH than the cytosol (68). A density plot (Fig. S1), made by Patrickios isoelectric points (69), of the human mitochondrial proteome (MitoCarta2.0 [5]) showed that mitochondrial proteins on average have a higher pI than the remainder of the proteome. Isoelectric points for the P. falciparum proteome were calculated using the Patrickios algorithm (70) and directly used as pI scores.

10.1128/mSphere.00614-21.4 FIG S1 Human mitochondrial proteins on average have a slightly higher isoelectric point (pI) than the nonmitochondrial remainder of the human proteome. Density plots over the Patrickios pI retrieved from a proteome isoelectric point database (69) of the human mitochondrial proteome (top) (5) and the nonmitochondrial remainder of the proteome (bottom). Red dotted lines indicate means. Download FIG S1, EPS file, 0.6 MB. Copyright © 2021 van Esveld et al. 2021 van Esveld et al. https://creativecommons.org/licenses/by/4.0/ This content is distributed under the terms of the Creative Commons Attribution 4.0 International license.

### (iii) Bayesian integration to predict mitochondrial proteins.

Using the eight data sets described above and the gold standard evaluation sets, a mitochondrial score for each P. falciparum reference protein was calculated. This score is the logarithm of the odds that a protein is localized in the mitochondrion relative to the protein being localized somewhere else in the cell. For categorical data (mitochondrial ortholog and apicoplast targeting signal), the data set is separated into bins that represent the two categories. Continuous data (the other six data sets) were binned in a systematic way using a custom python script (https://github.com/JordyCoolen/Binning) (settings: –bins 0 –score 1). In short, this script optimizes for each data set the distribution of the bins such that the sum of log ratio scores of all bins combined (see below) is close to zero, as this will result in the best separation of positive and negative bins. The number of bins was varied to a maximum of five to achieve the most optimal separation without expanding the number such that there are too few proteins per bin to obtain reliable estimates of the log odds of that bin.

The fractions of gold standard proteins per bin are determined. The log ratio of these fractions determines the score for all other proteins in that respective bin. The mitochondrial score is based on the sum of log ratios of the individual data sets and is calculated as follows:

$$\text{mitochondrial score }={\text{ log}}_{2}\left(\frac{P_{\text{mitochondrial}}}{P_{\text{nonmitochondrial}}}\right)+\overset{n}{\underset{i=1}{\sum }}{\text{log}}_{2}\left(\frac{P\left({\text{data}}_{i}|\text{mitochondrial}\right)}{P\left({\text{data}}_{i}|\text{nonmitochondrial}\right)}\right)$$

with

$$\frac{P({\text{data}}_{i}|\text{mitochondrial})}{P\left({\text{data}}_{i}|\text{nonmitochondrial}\right)}=\frac{{\text{mitochondrial\_pos}}_{i}}{{\text{mitochondrial\_neg}}_{i}}$$

where mitochondrial_pos and mitochondrial_neg are the fractions of the positive and negative gold standard genes in sample *i*, respectively. If no gold standard genes were found in a certain bin, that positive or negative gold standard fraction was set to 0.5/(total number of negative set genes in the complete data set) to prevent division by zero and allow calculation of the log ratio. *O*_prior_, calculated as log_2_(*P*_mitochondrial_/*P*_nonmitochondrial_), is based on the estimation that 536 proteins of the 5,357 protein-coding genes (∼10%) encode a mitochondrial protein. This is a conservative estimate compared to those for single-cell species like Saccharomyces cerevisiae, with 16% mitochondrial proteins (71), or the more closely related T. gondii, where 15% of proteins that could confidently be mapped are mitochondrial (23), while compared to all proteins that were mapped in that data set, the percentage of mitochondrial proteins is 10%. Note that *O*_prior_ affects only the score per protein; it does not affect the relative ranking of potential mitochondrial proteins.

To assess the performance of the integration, a false discovery rate (FDR) was calculated. As this FDR depends on gold standard genes and as the ratio of gold positives to negatives is not similar to the ratio of mitochondrial protein coding genes to nonmitochondrial coding genes in the genome, the FDR was corrected (cFDR) using the following formula:

$$\text{cFDR}=\frac{1\;-\text{ specificity}}{1\;-\text{ specificity }+\text{ sensitivity }\times \;O_{\text{prior}}}$$

### (iv) Cross-validation.

To assess the ability of the integrated predictor to discriminate known mitochondrial proteins from nonmitochondrial proteins, 10-fold cross-validation was performed. The gold standards (both negative and positive) were subsampled 10 times, thereby creating 10 sets of 9/10 of the gold standard genes. Each gold standard gene was left out once in one of the 10 sets. Data integration was performed with each of these 10 sets, and the ranks of the 1/10 omitted gold standard genes were retrieved. The 10-fold cross-validated receiver operating characteristic (ROC) curve was constructed based on those ranks.

### (v) Intraspecies homologs.

The human mitochondrial proteome has expanded since the divergence from yeast, mainly due to gene duplications that create mitochondrial paralogs (48). It is therefore interesting to see if the predicted P. falciparum mitochondrial proteins have intraspecies homologs and whether we can identify mitochondrial gene families. With HHpred (28), using default settings and a 1E−5 E value cutoff, the homologs within the genome of P. falciparum were determined and are included as a column in Table S1.

### (vi) Validation of candidate proteins.

To validate the predictions, we selected 14 genes with unusual or novel mitochondrial functions within the top 295 genes that fell within the 25% cFDR cutoff in the initial data integration. Note that during the project, the number of predicted mitochondrial proteins increased to 445 by including better orthology prediction and a better data set of likely apicoplast proteins. Of these 14 genes, 13 were selected based on maximum transcript levels during asexual blood-stage development of P. berghei strain ANKA cl15cy1 that exceeded 100 fragments per kilobase per million reads (FPKM) (72). In addition, we selected one particularly interesting gene, PF3D7_1004900, that had a lower transcript level. We used experimental genetics (73) to generate P. berghei lines expressing 3×HA-tagged copies of the selected proteins and colocalized them with an established mitochondrial marker (17) to determine their subcellular localization by immunofluorescence microscopy. Details on plasmid construction (successful for 13 of 14 targets, excluding PF3D7_1142800) and the generation of the lines can be found in the supplemental material. To assess colocalization, the freshly harvested transgenic parasites were allowed to settle on a poly-l-lysine-coated coverslip for 10 min and then fixed with 4% electron microscopy (EM)-grade paraformaldehyde (Fisher Scientific) and 0.0075% EM-grade glutaraldehyde (Sigma-Aldrich) in microtubule stabilizing buffer (MTSB; 10 mM MES [morpholineethanesulfonic acid], 150 mM NaCl, 5 mM EGTA, 5 mM glucose, 5 mM MgCl_2_ [pH 6.9]) for 20 min (74). Next, parasites were permeabilized with 0.1% Triton X-100 in phosphate-buffered saline (PBS) for 10 min. Samples in which we imaged mOrange and mCherry were hereafter stained with DAPI (4′,6-diamidino-2-phenylindole; 1:300) for 15 min and mounted on a microscope slide with Vectashield (Vector Laboratories). All other samples were blocked in 3% fetal calf serum (FCS)-PBS for 1 h. Samples were incubated overnight at 4°C with rat anti-HA (1:500; ROAHAHA; Roche) and chicken anti-GFP (1:1,000; Thermo Fisher) antibodies and for 1 h with 1:500 goat anti-rat Alexa Fluor 594 (Invitrogen)- and goat anti-chicken Alexa Fluor 488 (Thermo Fisher)-conjugated antibodies at room temperature. Nuclei were stained with DAPI (1:300) for 15 min, and coverslips were mounted on a microscope slide with Vectashield (Vector Laboratories). Samples were imaged with an SP8 confocal microscope (Leica; 63× oil lens) or LSM900 confocal microscope (Zeiss; 63× oil lens). Images were processed minimally and similarly with Fiji (75).

### Tools for data analysis.

Plots, statistics, and calculations were performed with the R statistical package (76) and the additional packages gplots (77), ggplot2 (78), ROCR (79), scales (80), and reshape (81). The separation of the data sets into bins was performed using a python script (see above).

10.1128/mSphere.00614-21.2 TABLE S2 Primers used to create plasmids and recombinant parasite lines. Download Table S2, DOCX file, 0.03 MB. Copyright © 2021 van Esveld et al. 2021 van Esveld et al. https://creativecommons.org/licenses/by/4.0/ This content is distributed under the terms of the Creative Commons Attribution 4.0 International license.

10.1128/mSphere.00614-21.3 TEXT S1 Details about WICCA and experimental procedures. Download Text S1, DOCX file, 0.03 MB. Copyright © 2021 van Esveld et al. 2021 van Esveld et al. https://creativecommons.org/licenses/by/4.0/ This content is distributed under the terms of the Creative Commons Attribution 4.0 International license.
