# Supplementary figures and images for: A Prioritized and Validated Resource of Mitochondrial Proteins in Plasmodium Identifies Unique Biology
Source: mSphere. 2021 Sep 8;6(5):e00614-21. doi: 10.1128/mSphere.00614-21 (PMC8550323; doi:10.1128/mSphere.00614-21)

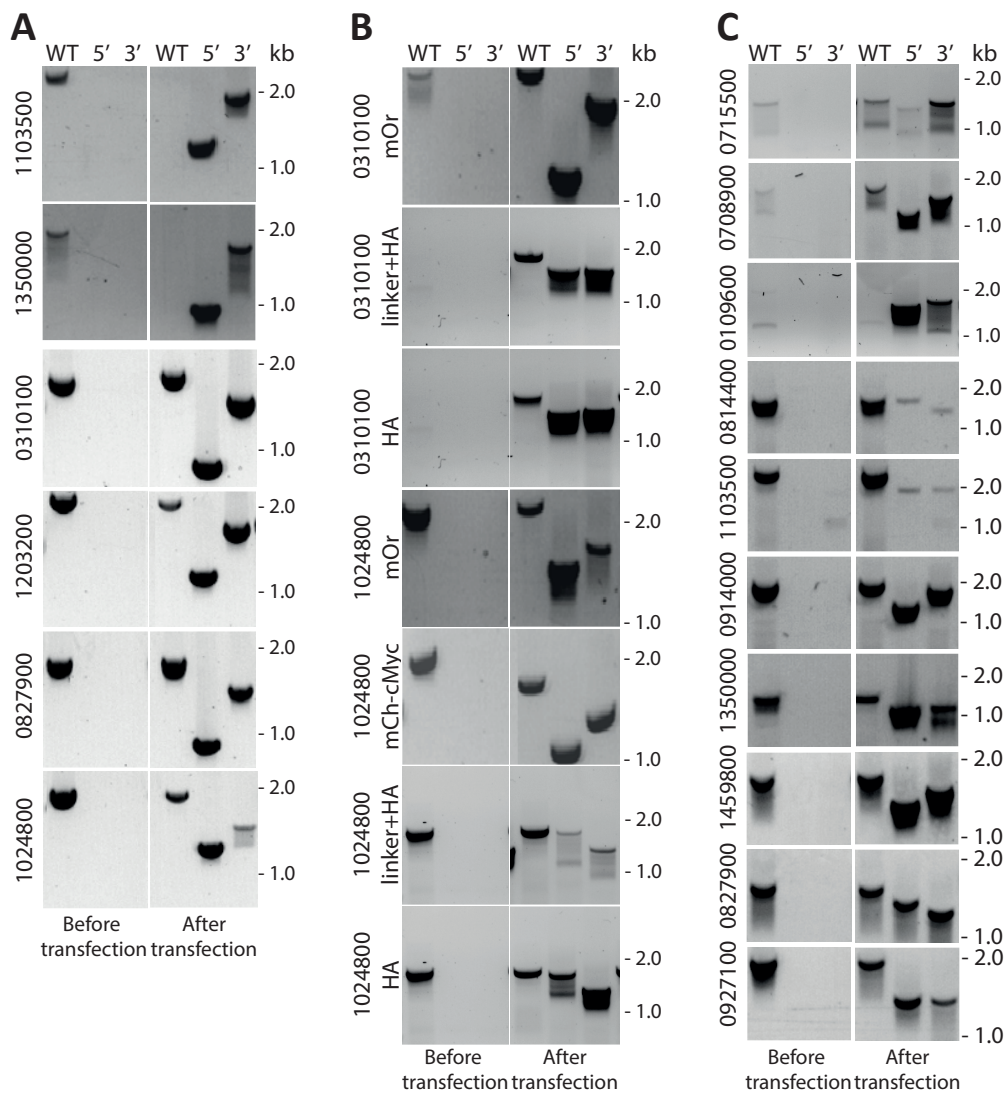

Supplement: FIG S6 [file msphere.00614-21-sf006.pdf]
