# Supplementary material for: A Prioritized and Validated Resource of Mitochondrial Proteins in Plasmodium Identifies Unique Biology
Source: mSphere. 2021 Sep 8;6(5):e00614-21. doi: 10.1128/mSphere.00614-21 (PMC8550323; doi:10.1128/mSphere.00614-21)
Supplement: TABLE S2 [file msphere.00614-21-st002.docx]

**Supplemental Table 2: Primers used to create plasmids and recombinant parasite lines.**

**General primers**

| Primer name | Sequence | Use |
| --- | --- | --- |
| MF-20i | GTAAAACGACGGCCAG | SQ |
| MR-invitrogen | CAGGAAACAGCTATGACC | SQ |
| R-HA-tag | CCGAAAAAGTTAAAATTAATTTAC | SQ |
| (a) F-Pb-pDHFR | ATGAAATACCGCTCCATTTTTCC | GT |
| (b) R-mFruit | GCCCTTGCTCACACCG | GT |
| (b) R-HA-2 | CCATGGTAAATGGATGATTCCCCTCTTTGC | GT |

**PBANKA_0715500: phosphoglucomutase-2**

| Primer name | Sequence | Use | WT^†^ | Int^§^ |
| --- | --- | --- | --- | --- |
| CT-0715500-F-EcoRI | TTAAATGAATTCAGAAAAAATCGAAATGATGAAAATTCC | TV | 543 |  |
| CT-0715500-R-SpeI | AAAATAACTAGTAAAATATGTAACACTATTGAAAGGAAGAT | TV |  |  |
| 3’-0715500-F-XhoI | TTTTAACTCGAGTGCACATTAGGTCGTTACTACG | TV | 512 |  |
| 3’-0715500-R-KpnI | TTTTATGGTACCTTTTTTTCCATTTTGCAATGAAGC | TV |  |  |
| (c) INT-0715500-F | TAAGTTACAGGAAAAAAAAGAAGGG | GT | 1325 | 1192 |
| (d) INT-0715500-R | CAATTGGTCAAAGGGAAGAGC | GT |  | 1288 |

**PBANKA_0109600: ATP-synthase-associated protein**

| Primer name | Sequence | Use | WT^†^ | Int^§^ |
| --- | --- | --- | --- | --- |
| CT-0109600-F-EcoRI | TTTAAGAATTCATGGTGGTAGATTTTTCATTTTGCC | TV | 744 |  |
| CT-0109600-R-SpeI | AATTTAACTAGTTTTCGTTGCGGGTATTAGCA | TV |  |  |
| 3’-0109600-F-XhoI | AAATTTCTCGAGTTAACAACATGAAAACAAACTCATGG | TV | 494 |  |
| 3’-0109600-R-KpnI | AATTAAGGTACCCAAATTTCACCATATCAACCGC | TV |  |  |
| (c) INT-0109600-F | GGTAAGCAGCATCCAAAATGG | GT | 1940 | 1448 |
| (d) INT-0109600-R | TTTGCCATGGGTTCATAGAGG | GT |  | 1644 |

**PBANKA_1024800: ATP synthase-associated protein**

| Primer name | Sequence | Use | WT^†^ | Int^§^ |
| --- | --- | --- | --- | --- |
| CT-1024800-F-EcoRI | TTTATGAATTCATGAAATTAACAACCTGATGAAATGC | TV | 604 |  |
| CT-1024800-R-SpeI | TAATTAACTAGTCCATTTATAAGCTACAAAATTTTCCTTAA | TV |  |  |
| 3’-1024800-F-XhoI | AAATTTCTCGAGAGCATATGGAATAAAATGATTTCTGC | TV | 567 |  |
| 3’-1024800-R-KpnI | AATTATGGTACCCGCTACCAAATTAATCAACCG | TV |  |  |
| (c) INT-1024800-F | TAAGCAAGTGTAAAAATATAGAAAACG | GT | 1607 | 1566/1063/1062*/1534**/1062*** |
| (d) INT-1024800-R | GGTTATAAAAAGAGACAATGTTGAGG | GT |  | 1226 |

**PBANKA_0310100: conserved protein, unknown function**

| Primer name | Sequence | Use | WT^†^ | Int^§^ |
| --- | --- | --- | --- | --- |
| CT-0310100-F-EcoRI | ATAATTGAATTCCGATTTAAATTTCCAGGGTTTTGG | TV | 791 |  |
| CT-0310100-R-SpeI | TTTTATACTAGTTTTAATTAATTCAATATATTTTTCATATGATGCA | TV |  |  |
| 3’-0310100-F-XhoI | AAAATTCTCGAGCATGTTCAGGTAAAAATGTCAATGTAC | TV | 541 |  |
| 3’-0310100-R-KpnI | TTTTATGGTACCTACTTGCATGTTCTTGAACTAGG | TV |  |  |
| (c) INT-0310100-F | AATTTTACCAGTCGAACAAGAACC | GT | 1713 | 1383/877/877*/1351** |
| (d) INT-0310100-R | ATAAATAACGAAATATAGAATCAAGTTTCC | GT |  | 1411 |

**PBANKA_0708900: conserved protein, unknown function**

| Primer name | Sequence | Use | WT^†^ | Int^§^ |
| --- | --- | --- | --- | --- |
| CT-0708900-F-EcoRI | TTTAAAGAATTCGACGAAGATGGTAAAGAAGAACC | TV | 493 |  |
| CT-0708900-R-SpeI | AAATTAACTAGTTTCATGAAAGGACTTTCTATATTCTTC | TV |  |  |
| 3’-0708900-F-XhoI | TTAATTCTCGAGGCCATTATTTCAATCAGCAAGC | TV | 549 |  |
| 3’-0708900-R-KpnI | AATTAAGGTACCTTATGTTTAGACGAAGACCAAATCC | TV |  |  |
| (c) INT-0708900-F | TAATTTTAGCTATTTCCAAAGGTATAGG | GT | 1625 | 1111 |
| (d) INT-0708900-R | CCTCACACCTCCAACGATTAC | GT |  | 1321 |

**PBANKA_1459800: conserved *Plasmodium* protein – unknown function**

| Primer name | Sequence | Use | WT^†^ | Int^§^ |
| --- | --- | --- | --- | --- |
| CT-1459800-F-EcoRI | TTTAAAGAATTCGAATCCGGAGAAATACCACTACC | TV | 522 |  |
| CT-1459800-R-SpeI | TTTTAATACTAGTTACTTTAATCCATGATATTTTGTCATCAATC | TV |  |  |
| 3’-1459800-F-XhoI | ATTTTACTCGAGATTTTGAATAGTTCTCATTTGAGGG | TV | 698 |  |
| 3’-1459800-R-KpnI | AAAATTGGTACCTTGCCAATGAAATAATATCAGTAATACC | TV |  |  |
| INT-1459800-F | CGATAAAATTGATAGAAGTTAACCCC | GT | 1627 | 1262 |
| INT-1459800-R | CATCCAACTGATCACATAGTGC | GT |  | 1427 |

**PBANKA_0814400: conserved *Plasmodium* protein, unknown function**

| Primer name | Sequence | Use | WT^†^ | Int^§^ |
| --- | --- | --- | --- | --- |
| CT-0814400-F-EcoRI | TTTAAAGAATTCGGCAAATTAAACAAGGTAGATTGG | TV | 547 |  |
| CT-0814400-R-SpeI | TTAAATACTAGTTTTTTTTCTTAAAGTTGCTAATAATGATG | TV |  |  |
| 3’-0814400-F-XhoI | TTTTAACTCGAGTTTATTATTAATTTATATTTTTATTTTCAACTAGC | TV | 554 |  |
| 3’-0814400-R-KpnI | TTTAATGGTACCGCCAACTTTTAAAGCACAACG | TV |  |  |
| (c) INT-0814400-F | TGTATGTATATGCATGCTTGTATGG | GT | 1457 | 1264 |
| (d) INT-0814400-R | GCCTATATCCAATCCTATTATCAATGG | GT |  | 1196 |

**PBANKA_1203200: conserved protein, unknown function**

| Primer name | Sequence | Use | WT^†^ | Int^§^ |
| --- | --- | --- | --- | --- |
| CT-1203200-F-EcoRI | ATAAAAGAATTCATGTAAAATGCTTAATGAAATATGAAGG | TV | 664 |  |
| CT-1203200-R-SpeI | TATTAATACTAGTTTTTTTAATTCTAGGTTTGTTTCTAGAAAG | TV |  |  |
| 3’-1203200-F-XhoI | AATTATCTCGAGATTTCATAGTTTGCTTAACTTCAAAG | TV | 478 |  |
| 3’-1203200-R-KpnI | ATTATAGGTACCATAATGAACCATGGAATATAATAAATGATTC | TV |  |  |
| (c) INT-1203200-F | AATACAATGTGAAATTTTGCTTTTTTCC | GT | 1802 | 1511/1002 |
| (d) INT-1203200-R | ATAATATTATAAGCCCATTTAGACTTTTAG | GT |  | 1435 |

**PBANKA_0827900: conserved protein, unknown function**

| Primer name | Sequence | Use | WT^†^ | Int^§^ |
| --- | --- | --- | --- | --- |
| CT-0827900-F-EcoRI | TTATATGAATTCTTTGGAGGGGAAAATGTCGAG | TV | 555 |  |
| CT-0827900-R-SpeI | AATAATACTAGTCTGATTTCCCTTGACTATTTCAAG | TV |  |  |
| 3’-0827900-F-XhoI | AAATTACTCGAGTGTTGAAAAAGTCCCACCAAGC | TV | 432 |  |
| 3’-0827900-R-KpnI | TATATTGGTACCCCAGATGAAATGTTTTCTCTCTCC | TV |  |  |
| (c) INT-0827900-F | CCATCAAAGCTCATATTTCAAAG | GT | 1467 | 1293/784 |
| (d) INT-0827900-R | TTAATGGTCAAGTACCGGATGC | GT |  | 1154 |

**PBANKA_1103500: conserved *Plasmodium* protein, unknown function**

| Primer name | Sequence | Use | WT^†^ | Int^§^ |
| --- | --- | --- | --- | --- |
| CT-1103500-F-EcoRI | TAATTTGAATTCCCCCACTATTAATGGATAAAGATGC | TV | 776 |  |
| CT-1103500-R-SpeI | AATTTAACTAGTTATATATCGGCCTAGTGTATTAAAGAA | TV |  |  |
| 3’-1103500-F-XhoI | TAATATCTCGAGTATTTTCTTTCATTTCGAACAAGTGG | TV | 710 |  |
| 3’-1103500-R-KpnI | AAATTTGGTACCTTTTTGCAGAGTAGCTTTATTCG | TV |  |  |
| (c) INT-1103500-F | ACTTCAAAAGACATTGGGATCG | GT | 2192 | 1596/1087 |
| (d) INT-1103500-R | CGATTTATTTATTACCTTTAACCATTTTGC | GT |  | 1570 |

**PBANKA_0927100: conserved protein, unknown function**

| Primer name | Sequence | Use | WT^†^ | Int^§^ |
| --- | --- | --- | --- | --- |
| CT-0927100-F-EcoRI | TTAAAAGAATTCTCAGCATTATTTCGGCAAGC | TV | 720 |  |
| CT-0927100-R-SpeI | TTATAAACTAGTAAAATAGTAATATTTAAAAAATTTAAAAATATTTTTTATATG | TV |  |  |
| 3’-0927100-F-XhoI | TATTTTCTCGAGTAATTTTTTTTCCATTGTTGGGTTTGC | TV | 556 |  |
| 3’-0927100-R-KpnI | TATATTGGTACCCCCACAGCATAGAGTTTTGC | TV |  |  |
| (c) INT-0927100-F | ATTTTTTAACGTGGATTTTTGTCCTG | GT | 1986 | 1360 |
| (d) INT-0927100-R | TATAAAGCAGTAAAGGAAATGTTCG | GT |  | 1352 |

**PBANKA_0914000: conserved protein, unknown function**

| Primer name | Sequence | Use | WT^†^ | Int^§^ |
| --- | --- | --- | --- | --- |
| CT-0914000-F-EcoRI | AATAATGAATTCGAATAATGAAAAAATACCATCAGAAACG | TV | 527 |  |
| CT-0914000-R-SpeI | TTATTTACTAGTTGTTTTCCTGCTTCTAAACACACC | TV |  |  |
| 3’-0914000-F-XhoI | TTAAATCTCGAGTATTGATCCATCTTCATTACTTAAACCT | TV | 764 |  |
| 3’-0914000-R-KpnI | ATTAATGGTACCAGGACAAAAATGAAAACGACAGC | TV |  |  |
| (c) INT-0914000-F | GATACTCGTTTTGATAATGAAATTTCG | GT | 1698 | 1207 |
| (d) INT-0914000-R | CTCGAATTATGCAGCGTACC | GT |  | 1575 |

**PBANKA_1350000: conserved protein, unknown function**

| Primer name | Sequence | Use | WT^†^ | Int^§^ |
| --- | --- | --- | --- | --- |
| CT-1350000-F-EcoRI | TTTAAAGAATTCCAAATAGAAAAGGGATAAATACTGAGG | TV | 591 |  |
| CT-1350000-R-SpeI | AAATTAACTAGTAATTTTTTTCGCAAATTCTTCGAG | TV |  |  |
| 3’-1350000-F-XhoI | TTAATTCTCGAGTTTTACGTACCGCCATTTAACG | TV | 667 |  |
| 3’-1350000-R-KpnI | AATTAAGGTACCTGATAAAATTCCATATAAAAGTACCATGC | TV |  |  |
| (c) INT-1350000-F | TGTAAACTATAAAGCCATTTGAATATCC | GT | 1501 | 1263/754 |
| (d) INT-1350000-R | TTATGTTAATTTTTTTTTCTCTCAACTCC | GT |  | 1314 |

SQ: primers used for SeQuencing of the integrated locus or fluorescent cassette.

TV: primers used for creation of Tagging Vectors.

GT: primers used for GenoTyping after integration.

†: Expected PCR product size on wild-type *P. berghei* strain ANKA gDNA performed with forward (c) and reverse (d) primers.

§: Expected PCR product size on gDNA of successful transgenic parasites tagged with linker-3xHA / mOrange-3xHA / mOrange* / 3xHA** / mCherry-cMyc***.
