# Supplementary material for: A Prioritized and Validated Resource of Mitochondrial Proteins in Plasmodium Identifies Unique Biology
Source: mSphere. 2021 Sep 8;6(5):e00614-21. doi: 10.1128/mSphere.00614-21 (PMC8550323; doi:10.1128/mSphere.00614-21)
Supplement: TEXT S1 [file msphere.00614-21-s0001.docx]

**Details about WICCA and experimental procedures**

**WeIghted Co-expression Calculation tool for plAsmodium genes (WICCA).** WICCA was written in R [1] (v3.3.1). The raw supplemented materials of 83 microarray experiments (12 *P. berghei*, 2 *P. chabaudi*, 66 P*. falciparum and*, 3 *P. vivax*) were retrieved from the Gene Expression Omnibus (GEO) [2] on 14 December 2016. The GEO ID’s of the datasets can be found in the wicca tool (https://wicca.cmbi.umcn.nl/). The raw data was further processed using the R package limma [3] to correct for background noise and to perform a quantile normalization to remove systematic bias. Technical replicates were averaged and probe identifiers were re-mapped to the up-to-date gene identifiers via the platform’s own annotation libraries and the PlasmoDB website [4]. As we want to use all 83 datasets for each of the four species mentioned above, the ortholog group information from PlasmoDB [4] was used to remap each dataset to the other three species. Pearson’s correlation coefficients were pre-calculated for every gene combination in all datasets as a measure of co-expression. The mean of these coefficients per dataset was calculated and used as background co-expression signal.

The user can enter a list of query genes for WICCA to calculate their co-expression and find potentially other genes of interest that are co-expressed with the input genes. The Pearson correlation between each gene and each query gene was calculated for every dataset, background co-expression signal was subtracted. The mean of these values per gene in each dataset was taken to obtain one background-corrected value per gene for each dataset.

WICCA assumes that the query genes come from a common pathway or share a functional relationship and therefore the pair-wise correlation between these query genes influences the weight that each of the 83 datasets ultimately get. A higher weight should be given to datasets that show high correlation between the query genes and less weight to datasets that do not. Weighing was performed by calculating a weighing score (ω_d_) for each dataset with the following formula:

$$\omega_{d}= \frac{\sum_{1}^{i} \sum_{1}^{j} r_{x_{1..i,}y_{1..j}}}{k^{2}-k}$$

Where *r* is the Pearson’s correlation coefficient of the expression values of gene *x* and *y*, *k* is the number of query genes, with *i* and *j* equal in size to *k* used here to distinguish between different combinations of query genes. The sum of the weights of all datasets is set to 1. The calculated weight for each dataset thus ranges between 0 and 1 and is a measure of how well the entered query genes co-express with each other within that particular dataset.

For each dataset the weighing score is multiplied by the calculated background-corrected Pearson correlation values. The sum of all weighted co-expression values associated with a gene across all datasets was calculated to obtain one final weighted gene-specific co-expression score. In the final results genes are ranked on this weighted value, with genes showing high co-expression with the query genes occurring at the top of the ranking.

**Evolutionary inference network setup.** In order to calculate the CLIME scores from the 138 species listed in the database, we used a multilayer perceptron (MLP) with two hidden layers, with 16 and 4 hidden nodes. For node activation, the softplus function was used. As the network output, two scores were calculated; a positive and negative score (for resembling the positive or the negative set, respectively). The final score was calculated as positive/(positive + negative) to represent a relative score.

Using the alternative positive/negative set as training data, the error was back-propagated through the network using the gradient descent method, adjusting the weights in between layers. 1280 iterations were performed. The resulting network was used to calculate scores for the testing data, which were later evaluated using 4-fold cross-validation. The area under the mean receiver-operator curve was 0.76+0.05, indicating that the CLIME scores are useful predictors for mitochondrial localization. This evaluation was performed independently of the Bayesian 10-fold cross-validation.

**Generation of tagging plasmids.** The initial vector including an hDHFR drug-selectable cassette, a mitochondrial GFP marker cassette, and a mOrange-3xHA tag with spacer sequence was obtained through triple ligation of elements from three different vectors. The plasmid backbone was derived from the pBAT vector (GenBank JX099571 [5]) digested with XhoI and EcoRI (2.1 kb), the drug-selectable and mitochondrial GFP marker cassettes were derived from the mitochondrial co-localization vector [6] digested with PvuII and XhoI (6.9 kb), and the linker-mOrange-3xHA tag was synthesized (GeneArt/Life Technologies) and digested with EcoRI and PmlI (1.3 kb). For testing of the effect of different tags on proteins localization, the mOrange-3xHA tag was replaced by the original mCherry-3xMyc tag, a mOrange-only tag, and two 3xHA tags, with and without linker sequence. Gene-specific 5’ and 3’ homology regions containing the carboxy-terminal (CT) ends and 3’ untranslated regions (UTR) were amplified from *P. berghei* strain ANKA gDNA and cloned into the respective plasmids, such that the tag was fused in frame with the protein coding sequence (Table S3, TV). All constructs were verified by commercial Sanger sequencing. Fig. S3 depicts a schematic overview of the tagging strategy.

**Generation of recombinant parasite lines.** The transfection plasmids targeting PBANKA_0715500 were linearized using AhdI and PvuI, all other plasmids were linearized using ApaLI and AhdI. Transfections were performed as described previously [7]. When parasitemias reached 1%, typically at days 6-9 after transfection, blood of the infected mice was harvested to make samples for microscopy and isolate gDNA for genotyping. Correct integration of the transfected vector was assessed by diagnostic PCR (Fig. S6 and Table S2).

1. R Core Team, *R: A Language and Environment for Statistical Computing*. 2015, R Foundation for Statistical Computing: Vienna, Austria.

2. Barrett, T., S.E. Wilhite, P. Ledoux, C. Evangelista, I.F. Kim, M. Tomashevsky, et al. NCBI GEO: archive for functional genomics data sets--update*.* Nucleic Acids Res 2013;41: D991-5.

3. Ritchie, M.E., B. Phipson, D. Wu, Y. Hu, C.W. Law, W. Shi, et al. limma powers differential expression analyses for RNA-sequencing and microarray studies*.* Nucleic Acids Res 2015;43: e47.

4. Aurrecoechea, C., J. Brestelli, B.P. Brunk, J. Dommer, S. Fischer, B. Gajria, et al. PlasmoDB: a functional genomic database for malaria parasites*.* Nucleic Acids Res 2009;37: D539-43.

5. Kooij, T.W., M.M. Rauch, K. Matuschewski. Expansion of experimental genetics approaches for Plasmodium berghei with versatile transfection vectors*.* Mol Biochem Parasitol 2012;185: 19-26.

6. Matz, J.M., C. Goosmann, K. Matuschewski, T.W.A. Kooij. An Unusual Prohibitin Regulates Malaria Parasite Mitochondrial Membrane Potential*.* Cell Rep 2018;23: 756-767.

7. Janse, C.J., B. Franke-Fayard, G.R. Mair, J. Ramesar, C. Thiel, S. Engelmann, et al. High efficiency transfection of Plasmodium berghei facilitates novel selection procedures*.* Mol Biochem Parasitol 2006;145: 60-70.
